# DCLRE1B promotes tumor progression and predicts immunotherapy response through METTL3-mediated m6A modification in pancreatic cancer

**DOI:** 10.1186/s12885-023-11524-8

**Published:** 2023-11-07

**Authors:** Lincheng Li, Fei Wang, Zhaoda Deng, Gong Zhang, Lin Zhu, Zhiming Zhao, Rong Liu

**Affiliations:** 1 Faculty of Hepato-Pancreato-Biliary Surgery, the First Medical Center, Chinese PLA General Hospital, Beijing, China; 2 Department of Surgery, Second Mobile Corps Hospital of Chinese People’s Armed Police Force, Wuxi, China

**Keywords:** DCLRE1B, Pan-cancer, Immunotherapy, m6A, Pancreatic cancer

## Abstract

**Background:**

DCLRE1B is a 5’-to-3’ exonuclease, which is involved in repairing ICL-related DNA damage. DCLRE1B has been reported to cause poor prognosis in a variety of cancers. Nonetheless, there is no research on DCLRE1B’s biological role in pan-cancer datasets. Thus, ascertaining the processes via which DCLRE1B modulates tumorigenesis was the goal of the extensive bioinformatics investigation of pan-cancer datasets in the present research.

**Methods:**

In our research, employing internet websites and databases including TIMER, GEPIA, TISIDB, Kaplan–Meier Plotter, SangerBox, cBioPortal, and LinkedOmics, DCLRE1B-related data in numerous tumors were extracted. To ascertain the association among DCLRE1B expression, prognosis, genetic changes, and tumor immunity, the pan-cancer datasets were examined. The DCLRE1B’s biological roles in pancreatic cancer cells were ascertained by employing wound healing, in vitro CCK-8, and MeRIP-qPCR assays.

**Result:**

According to the pan-cancer analysis, in numerous solid tumors, DCLRE1B upregulation was observed. Expression of DCLRE1B was found to be substantially related to the cancer patients’ prognoses. Similarly, expression of DCLRE1B exhibited substantial association with immune cells in several cancer types. DCLRE1B expression correlated with immune checkpoint (ICP) gene expression and impacted immunotherapy sensitivity. According to in vitro trials, DCLRE1B promoted PC cells’ proliferation and migration capacities. Also, according to GSEA enrichment analysis, DCLRE1B might participate in the JAK-STAT signaling pathway, which was confirmed by western blotting. In addition, we also found that the downregulation of DCLRE1B may be regulated by METTL3-mediated m6A modification.

**Conclusions:**

In human cancer, the overexpression of DCLRE1B was generally observed, which aided cancer onset and advancement via a variety of processes comprising control of the immune cells’ tumor infiltration. According to this study’s findings, in a few malignant tumors, DCLRE1B is a candidate immunotherapeutic and prognostic biomarker.

**Supplementary Information:**

The online version contains supplementary material available at 10.1186/s12885-023-11524-8.

## Introduction

The genetic material of the body, DNA, is frequently damaged by a variety of carcinogenic environmental conditions, elevating the likelihood of tumor onset [1]. DNA repair processes can restore minor DNA damage in healthy cells, which is critical for genomic stability [2]. Repairing damaged DNA is a complicated procedure involving numerous proteins and enzymes, among which nucleases have substantial influence.

DNA cross-link repair 1B (DCLRE1B/SNM1B) is a 5′-to-3′ exonuclease, which belongs to the metallo-β-lactamase (MBL) structural superfamily. DCLRE1B is critical in maintaining genome stability. DCLRE1B has been reported to promote telomere t-loop formation and protect the leading strand from inappropriate activation of non-homologous end joining (NHEJ). Besides, DCLRE1B has been interconnected to DNA lesion repair, specifically interstrand crosslinks (ICLs) and double-strand breaks (DSBs). Repair of DNA damage caused by ICL necessitates DCLRE1B, and its absence enhances sensitivity to agents that induce ICL, comprising mitomycin C and cisplatin C [3]. In addition, high expression of DCLRE1B gene in renal, liver and pancreatic cancers resulted in poor prognosis [4]. Karami et al*.* analyzed 24 GWAS data from five diverse types of cancer and found that rs974404 and rs12144215 were correlated with breast, prostate, colorectal, lung, and ovarian cancer risk [5]. All of the above research suggests that DCLRE1B could be a meaningful pan-cancer biomarker with prognostic and therapeutic value.

In our research, we explored the expression, prognosis, and immune infiltration of DCLRE1B in pan-cancer, including pancreatic cancer. In addition, the downstream influence of DCLRE1B knockdown was examined for the purpose of identifying the related signaling pathways involved in DCLRE1B. Furthermore, m6A methylation of DCLRE1B was also studied to further explore the related mechanism of DCLRE1B carcinogenesis.

## Method

### Data and software availability

The Cancer Genome Atlas (TCGA) (https://cancergenome.nih.gov/) and Gene Expression Omnibus (GEO) (https://www.ncbi.nlm.nih.gov/geo/) databases were employed for compiling the entire original data. Employing R 4.2.1, source data were combined, and we validated the website database investigation outcomes.

### Databases for analyzing the expression of DCLRE1B in human cancers

Employing GEPIA (http://gepia.cancer-pku.cn/) and TIMER (http://timer.comp-genomics.org/) databases, DCLRE1B expression was compared between human cancers and matched normal tissues. The TISIDB (http://cis.hku.hk/TISIDB/index.php) database was searched for ascertaining the relationships between DCLRE1B expression and stage or grade across human cancers. *P*-value < 0.05 indicated a significance level.

### Databases for analyzing the prognostic value of DCLRE1B in human cancers

In human cancers, DCLRE1B’s prognostic value was discovered via employing the GEPIA database and Kaplan–Meier Plotter database (http://kmplot.com/analysis/). In all cancer patients, employing the GEPIA database, the correlation between disease-free survival (DFS) or overall survival (OS) and DCLRE1B expression was examined. The median DCLRE1B expression was employed in the GEPIA database as a threshold value for the categorization of groups. The link between DCLRE1B expression and relapse-free survival (RFS) or OS in 21 distinct cancers was explored utilizing the Kaplan–Meier Plotter database, which categorized groups by automatically determining an ideal threshold value. When P-value was under 0.05, the prognostic value was deemed statistically substantial.

### Databases for analyzing the correlation between DCLRE1B expression and immune cell infiltration

Utilizing the SangerBox website (http://www.sangerbox.com/tool), the relationship between DCLRE1B expression and 22 immune cells in the tumor microenvironment (TME) of 33 human cancers was explored. The CIBERSORT method was used for assessing the relative and total abundance of 22 immune cell types. Furthermore, employing the TIMER databases, the correlation between DCLRE1B expression and cancer-associated fibroblast (CAF) was analyzed. In addition, the correlation between immune checkpoint (ICP) genes and DCLRE1B expression was also investigated.

### Database utilized for investigation of DCLRE1B genomic alterations

cBio Cancer Genomics Portal (c-BioPortal) (https://www.cbioportal.org/) compiled a multimodal cancer genomes dataset [6]. As a result, the c-BioPortal database was utilized for the investigation of DCLRE1B genetic variants in malignant tumors.

### Cell culture

American Type Culture Collection (Manassas, VA, USA) provided the human pancreatic ductal epithelial cell line HPDE6-C7 and pancreatic cancer cell lines BxPC-3, Capan-1, MIA PaCa-2, PANC-1, and SW1990. The cell lines were incubated in either RPMI 1640 or DMEM containing 10% fetal bovine serum (FBS, Gibco), and placed them in an environment containing 5% CO_2_ at 37℃.

### RNA transfection

Small interfering RNAs (siRNAs) against DCLRE1B and METTL3 were synthesized by Jintuosi (Wuhan) Biotechnology Co., Ltd. SW1990 and PANC-1 were transfected utilizing Lipofectamine 2000 (Invitrogen Corporation, Carlsbad, CA, USA) in accordance with the guidelines of the manufacturer. The siRNA sequences were as follows: DCLRE1B-siRNA: F:5′-GGAUCAAGAAGCAGUUGUUTT-3′, R:5′-AACAACUGCUUCUUGAUCCTT-3′; METTL3-siRNA-1: F:5′-GCUACCUGGACGUCAGUAUTT-3′, R:5′-AUACUGACGUCCAGGUAGCTT-3′; METTL3-siRNA-2: F:5′-GGUUGGUGUCAAAGGAAAUTT-3′, R:5′-AUUUCCUUUGACACCAACCTT-3′. After transfection for 24–48 h, the cells were collected for further RT-PCR and WB assays.

### Wound healing assay

Cell migration ability was assessed by the wound healing assay as described. Onto six-well plates, cells were plated for transfection with DCLRE1B siRNA or a negative control siRNA at a density of 3 × 10^5^ cells/well. And the confluent monolayer of cells was scratched with a wound using a 200 μl micropipette tip. Images were captured employing an inverted microscope at 0, 12, and 24 h after PBS washing. Cell migration rate was computed utilizing the following formula: (initial wound area – 12/24 h wound area) / initial wound area.

### Cell proliferation assay

Employing CCK-8 assay, cell proliferation was spotted. In a 96-well plate, cells were seeded at a 1,000 cells/well density and cultured in an environment containing 5% CO_2_ at 37 °C. After 1, 2, 3, and 4 d, a CCK-8 reagent at a volume of 10 μL was introduced into each well before placing them back into the cell culture incubator for additional two hours. Afterward, the absorbance was measured at 450 nm.

### Western blotting

RIPA lysis buffer (Beyotime) comprising 1% PMSF was employed to extract total cell proteins, after which the lysed proteins were centrifuged at 12,000 g at 4 °C for 10 min, along with subsequent protein quantification utilizing the BCA Protein Assay kit. Then, each protein sample was separated utilizing 10% SDS-PAGE and placed onto PVDF membranes (EMD Millipore, Billerica, MA, USA). Blockage of membranes was then performed in 5% nonfat milk, along with subsequent incubation with primary antibodies against: DCLRE1B (1:1000; orb258921, Biorbyt, UK), METTL3 (1:1000; 15,073–1-AP, Proteintech, USA), PD-L1 (1:1000; #13,684; Cell Signaling Technology, USA), Phospho-Stat3 (1:2000; #9145; Cell Signaling Technology, USA) at 4 °C for an overnight period. After three washes in TBST at 10-min intervals, the membranes were cultured at room temperature with the suitable horseradish peroxidase-labeled secondary antibody for 1.5 h. Protein signals were detected employing enhanced chemoluminescence (ECL) reagents.

### qRT-PCR detection

Extraction of total RNA was done employing the Trizol reagent (Invitrogen), as per the guidelines of the manufacturer. The cDNA was generated through reverse transcription of RNA samples with the aid of the RevertAid RT Kit (Thermo Scientific). And qPCR assays were then performed using RealStar Fast SYBR qPCR Mix (GenStar). Utilizing the StepOnePlus Real-Time PCR System (Applied Biosystems), gene amplification levels were measured. The mRNA’s relative expression was computed employing the 2^−ΔΔCt^ method, with internal control of 18S. Table S1 depicts the primers for RT-qPCR.

### Immunohistochemical staining

For immunohistochemistry (IHC), 5 μm thick sections were deparaffinized and rehydrated in xylene and a graded series of ethanol. Antigen retrieval was executed in a 10 mM sodium citrate buffer (pH 6.0) microwaved at 100 °C for 20 min. Sections were exposed to endogenous peroxide scavenging agents before being blocked with 5% normal goat serum and cultured at 4 °C overnight with antibodies against DCLRE1B (1:200; orb258921, Biorbyt, UK) and METTL3 (1:200; 15,073–1-AP, Proteintech, USA). After rinsing with PBS, the tissues were cultured at 37 °C for 40 min with an HRP-labeled secondary antibody and 3,3' -diaminobenzidine (DAB) for 5 min before being counterstaining for 30 s with hematoxylin.

### Methylated RNA immunoprecipitation-qPCR

Utilizing GenSeq® m6A MeRIP Kit (GenSeq, China), MeRIP-qPCR analysis was conducted as per the instructions of the manufacturer. Briefly, mRNA was fragmented for 6 min at 70 °C using RNA Fragmentation buffer. 3ug of fragmented RNA was saved for use as input RNA. Anti-m6A antibody was applied to immunoprecipitate fragmented RNA at 4 °C for 1 h. qPCR analysis was used to determine m6A enrichment.

### Statistical analysis

Data were expressed as mean ± SEM. Utilizing one-way ANOVA or two-tailed unpaired Student's t-test, statistical analyses were conducted for ascertaining significant variations (differences) across the data. Variations with *P*-values < 0.05 reached the significance level.

## Results

### DCLRE1B is substantially differentially expressed across tumor and normal tissues in human cancers

**Fig. 1:**
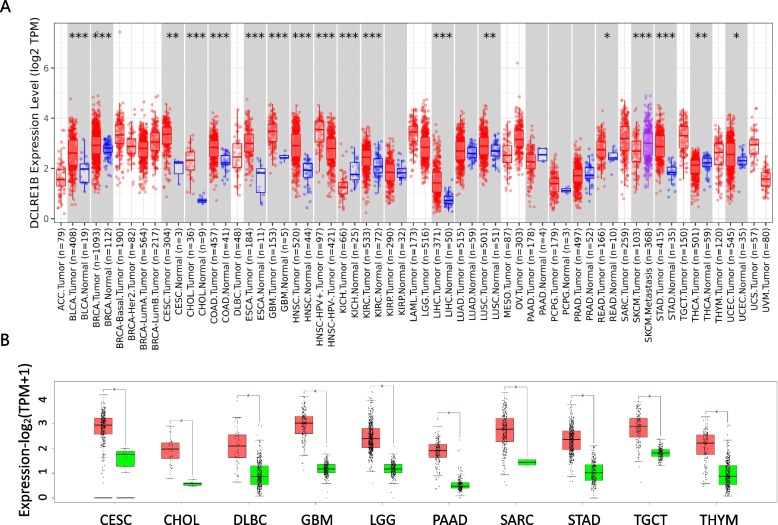
DCLRE1B is significantly differentially expressed in human cancers. **A** The expression of DCLRE1B in the TIMER database. **B** The expression of DCLRE1B in the GEPIA database. *P < 0.05; **P < 0.01; ***P < 0.001

**Fig. 2:**
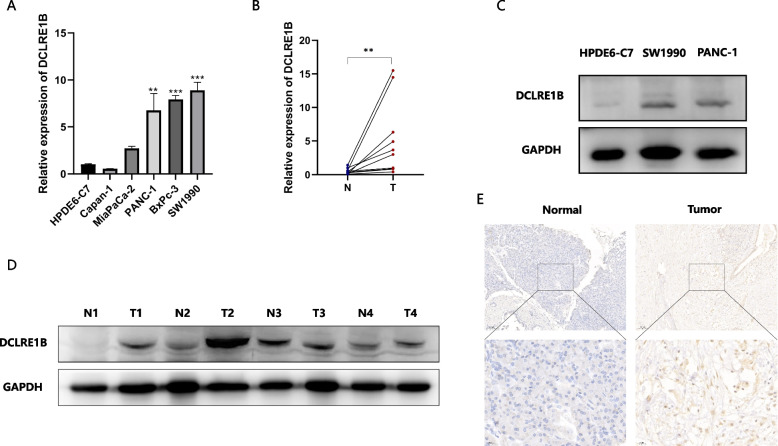
DCLRE1B is highly expressed in pancreatic cancer. **A**, **B** The mRNA expression of DCLRE1B in pancreatic cancer cell lines and tissues. **C**, **D** The DCLRE1B protein expression in pancreatic cancer cell lines and tissues. **E** IHC analysis for DCLRE1B in pancreatic cancer tissues. **P < 0.01; ***P < 0.001

According to the TIMER database, DCLRE1B expression was substantially enhanced in BLCA, BRCA, CESC, CHOL, COAD, ESCA, GBM, HNSC, KIRC, LIHC, LUSC, READ, STAD, and UCEC in comparison with adjacent normal tissue. DCLRE1B mRNA expression, nevertheless, was attenuated in KICH and THCA (Fig. 1A). The GEPIA database analysis results were available as supplementary results of cancers without paired normal tissues in the TIMER database. Moreover, according to the outcomes, the majority of cancer types had considerably greater DCLRE1B mRNA expression levels (Fig. 1B), which agreed with the findings derived based on the TIMER database. Subsequently, utilizing the TISIDB website, the correlations of DCLRE1B mRNA expression with tumor stage and grade among human cancers were ascertained. For the tumor stage, a significant positive correlation could be observed in ACC, KIRP, LIHC, and PAAD. In contrast, DCLRE1B expression was inversely linked to tumor stage in BRAC, CHOL, COAD, STAD, and TGCT (Supplementary Figure S1A). Correspondingly, higher DCLRE1B mRNA expression was linked to higher tumor grade in HNSC, KIRC, LGG, LIHC, and UCEC (Supplementary Figure S1B). Beyond this, we further verified these results in pancreatic cancer cells and tissues. As shown in Figs. 2A-B, DCLRE1B mRNA expression in pancreatic cancer cells and tissues was considerably greater compared to that in healthy pancreatic epithelial cells and tissues. In addition, the DCLRE1B protein’s high expression in pancreatic cancer was further confirmed as per western blotting and immunohistochemistry (Figs. 2C-E).

### Impact of DCLRE1B on proliferation and migration of PC cells

**Fig. 3:**
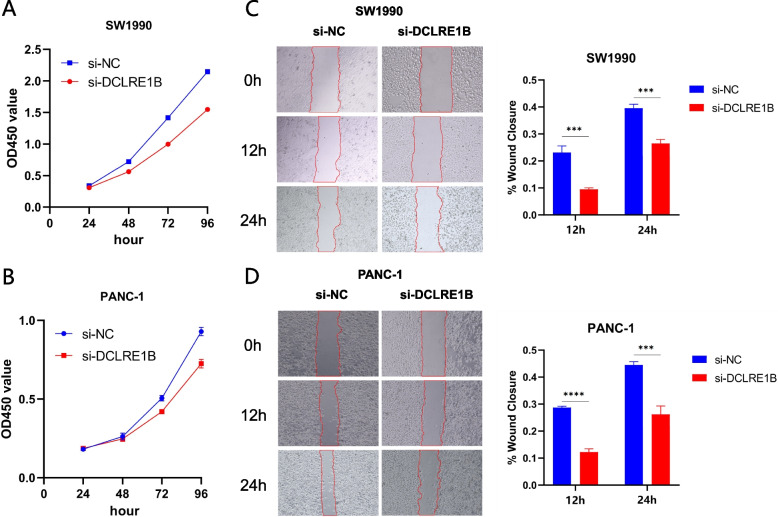
DCLRE1B promotes cell proliferation and migration in pancreatic cancer. **A**, **B** Cell viability of different groups of cells was measured by CCK8 assay in SW1990 and PANC-1 cells. **C**, **D** Cell migration ability of different groups of cells was detected by wound healing assays in SW1990 and PANC-1 cells. ***P < 0.001; ****P < 0.0001

To see how DCLRE1B affected PC, si-DCLRE1B was transfected in SW1990 and PANC-1 cells, respectively, to substantially lower DCLRE1B expression levels. According to CCK-8 assay findings, DCLRE1B knockdown substantially diminished cell proliferation in SW1990 and PANC-1 cells (Figs. 3A-B). The outcomes of wound healing revealed that silencing DCLRE1B could impair cell migration ability (Figs. 3C-D). Collectively, we could conclude that knocking down DCLRE1B dramatically reduced cell proliferation and migration as examined by CCK-8 and wound healing assay.

### DCLRE1B is a prognostic pan-cancer biomarker

**Fig. 4:**
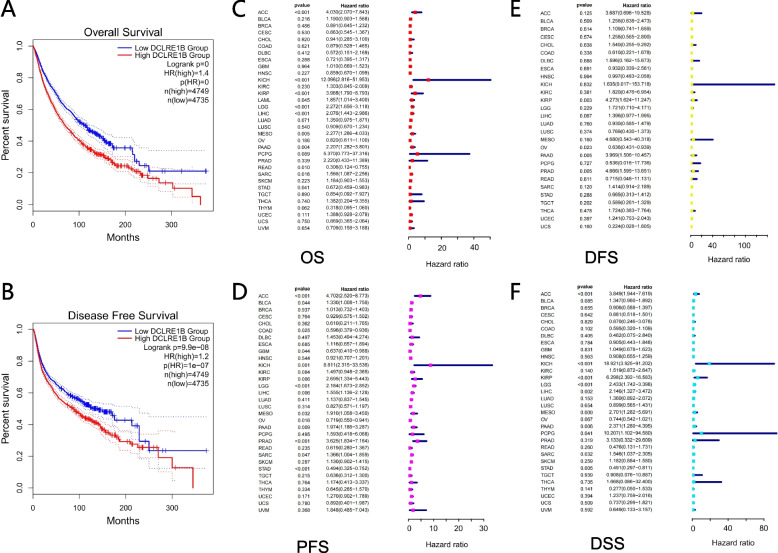
DCLRE1B is a prognostic biomarker in pan-cancer. **A**, **B** The correlation of DCLRE1B expression with OS and DFS across cancers by Kaplan–Meier analysis. **C**-**F** Forest plots of the association between DCLRE1B expression and OS, PFS, DFS, and DSS in 33 types of tumor

In human cancers, several databases were employed for evaluating the prognostic value of DCLRE1B expression. We discovered that, in GEPIA, the elevated DCLRE1B expression level was linked to the more unfavorable OS and DFS across cancers (Figs. 4A-B). The analysis of the Cox proportional hazards model showed that a positive relationship was evident between DCLRE1B expression and OS in ACC, KICH, KIRP, LAML, LGG, LIHC, MESO, PAAD, and SARC, whereas a negative relationship was discovered in READ and STAD (Fig. 4C). In terms of PFS, DCLRE1B expression had a protective role in COAD, GBM, OV, as well as STAD, however, it appeared to be a risk factor in ACC, BLCA, KICH, KIRP, LIHC, MESO, PAAD, PRAD and SARC (Fig. 4D). Regarding DCLRE1B and DFS, a substantial negative relationship was found in OV, while a positive relationship was observed in KIRP, PAAD, and PRAD (Fig. 4E). Furthermore, the DSS forest plot verified the protective function of DCLRE1B expression in STAD and its function as a risk factor in ACC, KICH, KIRP, LGG, LIHC, MESO, PAAD, PCPG, and SARC (Fig. 4F). Higher DCLRE1B expression was linked to poorer OS and RFS in LIHC and PAAD according to the Kaplan–Meier plotter database (Supplementary Figure S2A-B). The findings above exhibited a strong link between DCLRE1B expression and prognosis in several cancer types.

### Underlying association between DCLRE1B expression and immune infiltration

**Fig. 5:**
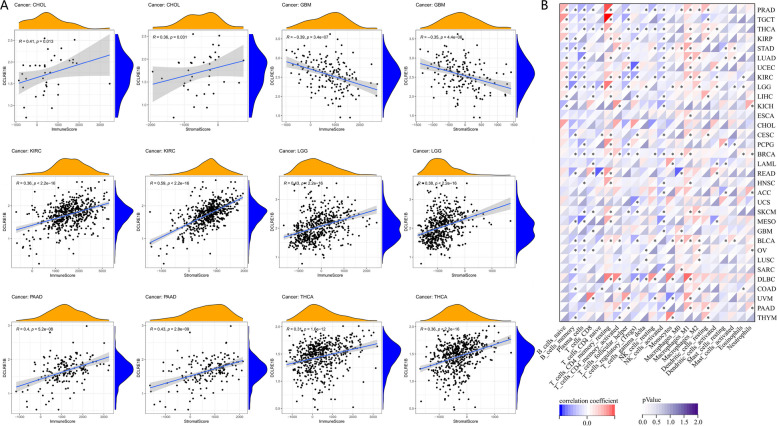
A strong connection between DCLRE1B expression and immune infiltration in multiple malignancies. **A** Significant correlations of DCLRE1B expression with immune score and stromal score were examined by ESTIMATE algorithm. **B** The relationship between DCLRE1B expression and immune cell infiltration level was analyzed in various tumors by Sangerbox online website. *P < 0.05

**Fig. 6:**
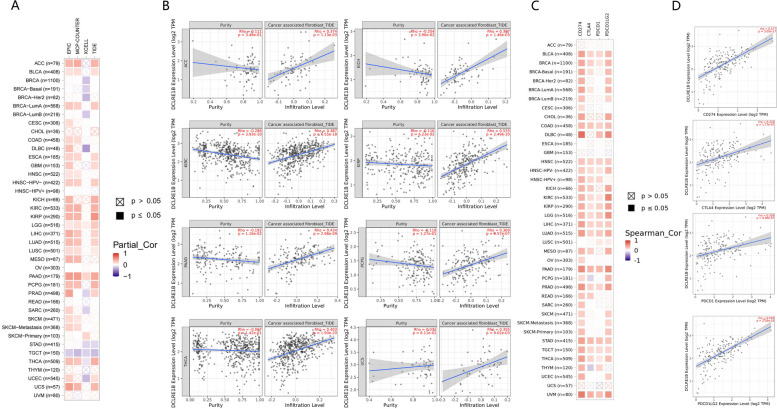
Associations of the DCLRE1B expression level with immune checkpoint genes. **A**, **B** The correlation of DCLRE1B expression and abundance of cancer-associated fibroblasts in pan-cancer. **C**, **D** The association between DCLRE1B expression and immune checkpoint genes was analyzed by TIMER database

**Fig. 7:**
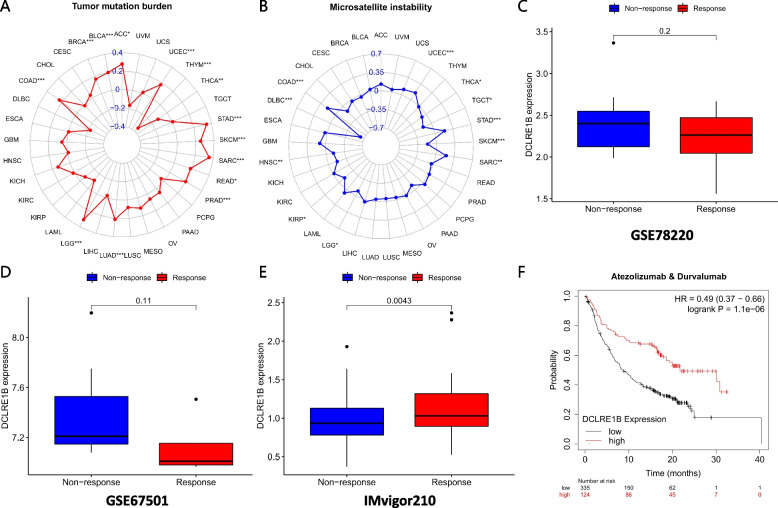
Relationship between DCLRE1B expression and immunotherapy. **A**, **B** The relationship between DCLRE1B expression and TMB as well as MSI was illustrated by a radar plot. **C**-**E** The correlation between DCLRE1B expression and immunotherapeutic response in GSE78220, GSE67501, and IMvigor210. **F** Kaplan–Meier analysis of the association between DCLRE1B expression and OS after treatment with Atezolizumab or Durvalumab. *P < 0.05; **P < 0.01; ***P < 0.001

The link between DCLRE1B expression and stromal score, immune score, and immune cell infiltration was investigated. Notably, DCLRE1B expression was positively associated with immune and stromal scores in CHOL, KIRC, LGG, PAAD, and THCA whereas it was negatively linked to GBM immune and stromal scores (Fig. 5A). The link between DCLRE1B expression and tumor-infiltrating immune cell (TIIC) levels in various tumors was then investigated employing the Sangerbox database. We could find that DCLRE1B expression showed a significant correlation with immune cells across multiple cancer types (Fig. 5B). For PAAD, a negative interrelation was confirmed between the DCLRE1B gene and NK cells, T follicular helper cells, and plasma cells, but DCLRE1B expression and CD4 + T cells, M1 macrophages, neutrophils, and DCs indicated a positive association. Cancer-associated fibroblasts (CAFs) substantially influence the advancement and therapy of cancer, according to several investigations [7, 8]. Therefore, in the TIMER database, we investigated the association between DCLRE1B and CAFs. According to the outcomes, DCLRE1B expression had a positive link to CAFs in ACC, KICH, KIRC, KIRP, PAAD, PCPG, THCA, and UCS using Tumor Immune Dysfunction and Exclusion (TIDE) algorithm (Figs. 6A-B), highlighting that DCLRE1B might be utilized as a target for treating these cancers. According to some findings, ICP genes have a significant impact on immunotherapy and immune cell infiltration [9]. The potential of DCLRE1B in immunotherapy was next investigated by examining the relationships between DCLRE1B expression and ICP genes like PD-1, PD-L1, PD-L2, and CTLA4 in human malignancies. It was discovered that DCLRE1B expression was positively associated with ICP genes in many types of cancer, especially in PAAD, in which the correlation coefficients of DCLRE1B with PD-1, PD-L1, PD-L2, and CTLA4 were 0.358, 0.577, 0.593 and 0.378, respectively (Figs. 6C-D). In addition, the correlations between DCLRE1B expression and TMB and MSI were also examined. According to the findings, DCLRE1B expression had a positive link to improved TMB in ACC, BLCA, BRCA, COAD, LGG, LUAD, PRAD, READ, SARC, SKCM, STAD, and UCEC, whereas DCLRE1B expression was negatively linked to TMB in THCA and THYM (Fig. 7A). Furthermore, a positive relationship between DCLRE1B expression and MSI was exhibited in COAD, SARC, STAD, and UCEC, while a negative relationship between DCLRE1B mRNA expression and MSI was discovered in DLBC, HNSC, KIRP, LGG, SKCM, TGCT and THCA (Fig. 7B). Furthermore, three pertinent independent immunotherapeutic cohorts (GSE67501, GSE78220, and IMvigor210) were examined in the current research. As shown in Figs. 7C-D, no substantial variation in DCLRE1B expression was noted across the groups with responder and non-responder in GSE67501 and GSE78220 cohorts. However, in the IMvigor210 cohort, patients with high DCLRE1B expression appeared to respond to immunotherapy more strongly (Fig. 7E). Consistent with this, patients with high DCLRE1B expression had longer survival after treatment with Atezolizumab or Durvalumab (Fig. 7F). Thus it can be seen that high DCLRE1B expression might predict good immunotherapy therapeutic efficacy, and DCLRE1B may serve as an ideal immunotherapeutic target in pan-cancer.

### Genetic alteration analysis of DCLRE1B in pan-cancer

**Fig. 8:**
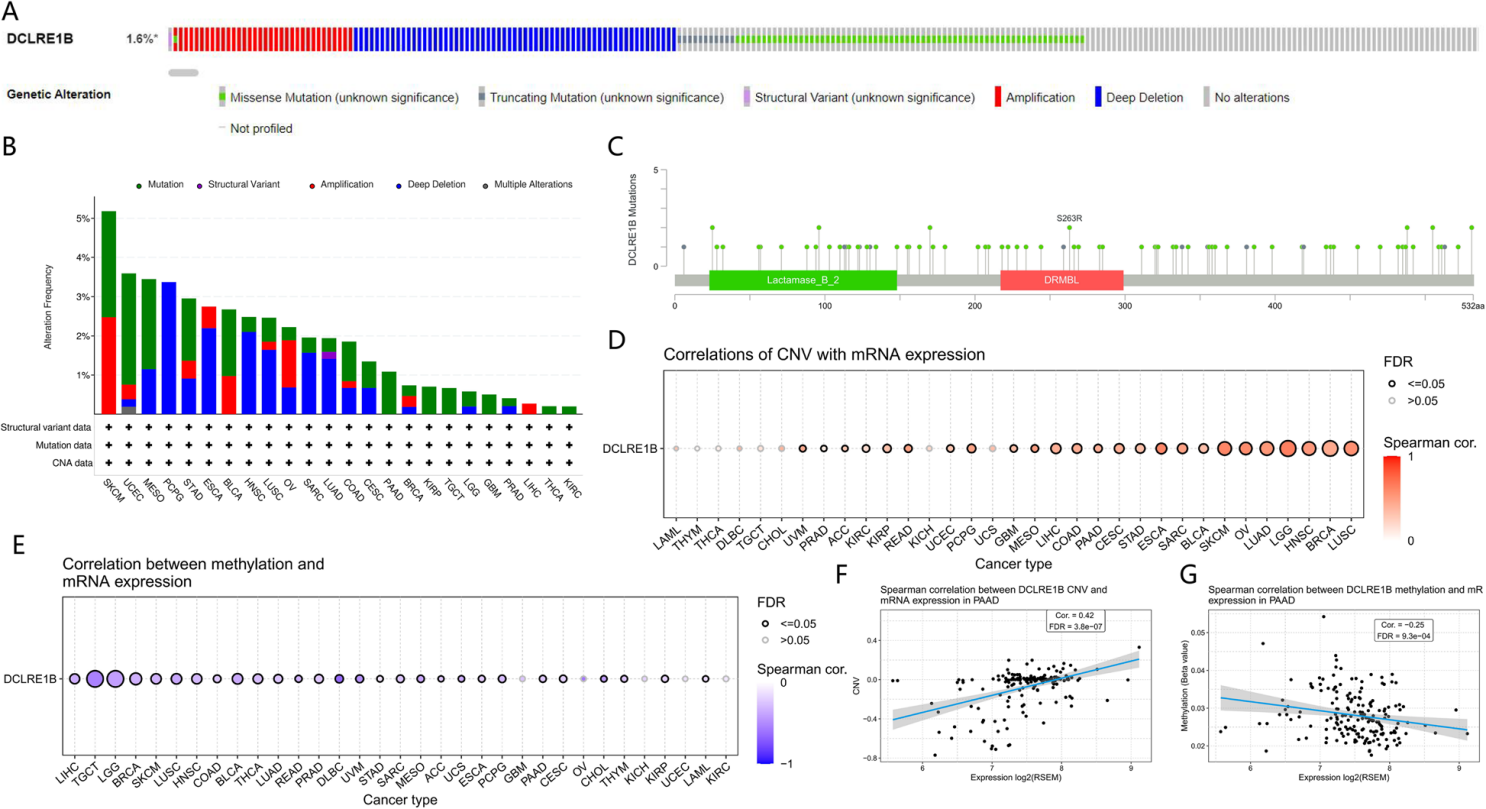
Characterization of genetic alterations in DCLRE1B. **A** General profile of genetic alterations in DCLRE1B. **B** Frequency of DCLRE1B mutations in different tumor types. **C** Types, sites and number of case with DCLRE1B genetic alterations in pan-cancer from cBioPortal. **D** The correlation of CNV with DCLRE1B expression in pan-cancer database. **E** The correlation between methylation and DCLRE1B expression in pan-cancer database. **F** The correlation of CNV with DCLRE1B expression in pancreatic cancer. **G** The correlation between methylation and DCLRE1B expression in pancreatic cancer

Utilizing the cBioPortal website, analysis of DCLRE1B genetic alterations was investigated. The finding revealed that 1.6% of cancer patients had DCLRE1B genetic abnormalities (Fig. 8A). And the frequent genetic alterations included deep deletion, missense mutation, and amplification. The maximum gene alteration rate of DCLRE1B was recorded for pan-cancer patients with uterine corpus endometrial carcinoma, skin cutaneous melanoma, mesothelioma and pheochromocytoma and paraganglioma with deep deletion (> 3%) as the primary type (Fig. 8B). The DCLRE1B gene modification types, sites, and case numbers were also displayed (Fig. 8C). In addition, a Spearman correlation between DCLRE1B CNV and mRNA was carried out. In LGG, SKCM, ESCA, OV, and LUSC, there existed a significant positive relationship between DCLRE1B CNV and mRNA expression. However, this link was insignificant in LAML, THYM, DLBC, THCA, KICH, CHOL, TGCT, and UCS (Fig. 8D). Investigations on the pan-cancer DCLRE1B methylation landscape also took place. With the exception of KICH, UCEC, OV, GBM, and KIRC, DCLRE1B methylation was exhibited to be substantially linked to DCLRE1B mRNA expression in the majority of cancers. Particularly, in DLBC, TGCT, UVM, CHOL, and LGG, the relevance is particularly obvious (Fig. 8E). Figures 8F-G showed the relationship between DCLRE1B CNV, methylation, and mRNA expression in pancreatic cancer.

### DCLRE1B and drug response

In our research, we discovered that DCLRE1B expression was favorably linked to medication response in patients treated with VT-464, ZM-336372, LSZ-102, Fenretinide, Cordycepin, AZD-9496, GDC-0810, PKM2 (9), Raloxifene and Curcumin. Furthermore, there exists a negative link between DCLRE1B expression and the anticancer drug SW-044248. Supplementary Figure S3 depicted the link between DCLRE1B expression and predicted drug response.

### METTL3-mediated m6A modification is associated with the downregulation of DCLRE1B in PAAD

**Fig. 9:**
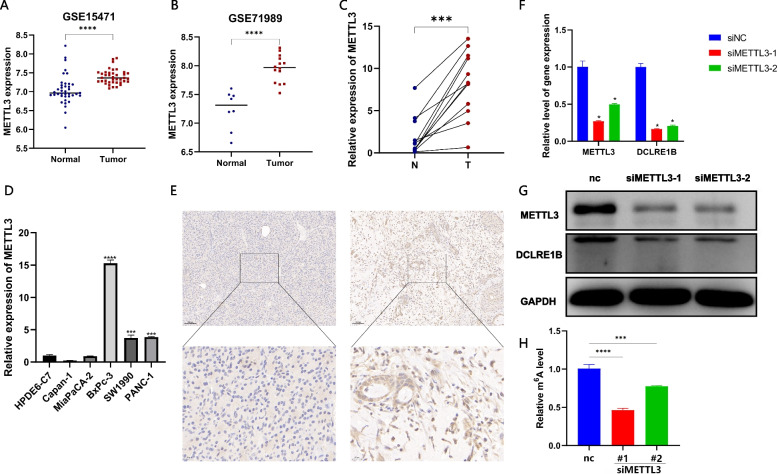
METTL3-mediated m6A modification is associated with the downregulation of DCLRE1B in PAAD. **A**, **B** The expression of METTL3 in public databases including GSE15471 and GSE71989. **C** The METTL3 expression in PC tissues by qRT-PCR. **D** The METTL3 expression in pancreatic cancer cell lines using qRT-PCR. **E** Immunohistochemistry analysis for METTL3 expression in PC tissues. **F** The mRNA expression of METTL3 and DCLRE1B after siMETTL3 transfection. **G** The protein level of METTL3 and DCLRE1B after siMETTL3 transfection by western blotting. **H** The m6A level of DCLRE1B after knockdown of METTL3. *P < 0.05; ***P < 0.001; ****P < 0.0001

We further analyzed the relationship between DCLRE1B and m6A-related genes. According to the findings, a substantial link existed between DCLRE1B and m6A modification (Supplementary Figure S4), and the underlying mechanism may need to be further explored. To explore whether the expression of DCLRE1B is regulated by m6A modification, we first predicted the possible m6A modification sites of DCLRE1B by the SRAMP database (http://www.cuilab.cn/sramp). The coding DNA sequences (CDS) of DCLRE1B were obtained from the NCBI database. The results of the SRAMP prediction analysis revealed that m6A modification sites were plentiful in DCLRE1B, demonstrating that DCLRE1B was very likely to be modified by m6A methylation (Supplementary Figure S5). In the m6A methyltransferase complex, which is primarily in charge of catalyzing the m6A modification of RNA molecules on N6-methyladenine, METTL3 is one of the most crucial proteins. METTL3 was discovered to be expressed at high levels in pancreatic cancer databases, according to our research, including GSE15471 and GSE71989 datasets (Figs. 9A-B). In clinical samples, the expression levels of METTL3 were then assessed. In comparison with paracarcinoma tissues, the METTL3 expression in PC patients was remarkably increased (Fig. 9C). Moreover, METTL3 expressions in pancreatic cancer cell lines were all remarkably rose (Fig. 9D), which was more remarkable in BxPc-3 cells. Immunohistochemistry assay also confirmed the up-regulated expression of DCLRE1B protein in pancreatic cancer tissues (Figs. 9E). After the knockdown of METTL3, we discovered that the DCLRE1B expression level was also substantially down-regulated by RT-qPCR and western blotting (Figs. 9F-G). Furthermore, MeRIP-qPCR analysis exhibited that the level of m6A-modified DCLRE1B was also substantially attenuated when DCLREB was knocked down by siRNA (Fig. 9H).

### DCLRE1B regulates cell function through the STAT3/PD-L1 pathway

**Fig. 10:**
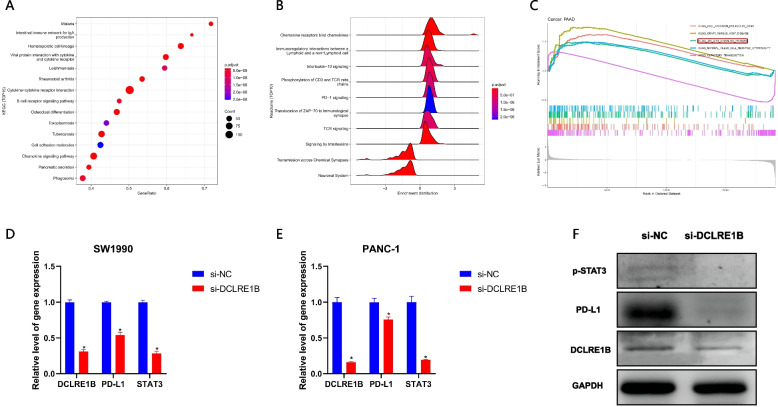
DCLRE1B regulates cell function through the STAT3/PD-L1 pathway. **A** KEGG pathway analysis for DCLRE1B in pancreatic cancer. **B** Reactome pathway analysis for DCLRE1B in pancreatic cancer. **C** GSEA enrichment analysis for DCLRE1B. **D**, **E** The mRNA expression of DCLRE1B, PD-L1, and STAT3 after siDCLRE1B transfection in SW1990 and PANC-1 cells. **F** The protein level of DCLRE1B, PD-L1, and p-STAT3 after knockdown of DCLRE1B by western blotting. *P < 0.05

According to the findings, DCLRE1B was significantly associated with cancer immunity and prognosis. Following that, we constructed a PPI network for DCLRE1B using the online GeneMANIA tool, which is displayed in Supplementary Figure S6. DCLRE1B demonstrated significant physical interactions with TERF2, SPAG5, KANSL2, TXLNA, and FNBP1L. To illustrate the potential function of DCLRE1B in cancer, we conducted KEGG and Reactome pathway analysis using PAAD as an example. We found that DCLRE1B was significantly enriched in the oncogenic and immune-related signaling pathways including the B cell receptor signaling pathway, chemokine signaling pathway, cytokine-cytokine receptor interaction, PD-1 signaling, and TCR signaling (Figs. 10A-B). These findings suggested that DCLRE1B expression might be crucial in human malignancies via regulating the tumor immune microenvironment. Furthermore, GSEA enrichment analysis showed that DCLRE1B may participate in JAK-STAT signaling pathway (Fig. 10C). Combined with the significant positive correlation between DCLRE1B and PD-L1, we speculated that DCLRE1B might regulate cell function through the STAT3/PD-L1 signaling pathway, which was confirmed by PCR and WB experiments in our research (Figs. 10D-F).

## Discussion

DNA interstrand cross-linking (ICL) is one of the most harmful types of DNA damage, which can be caused by mutagens such as cisplatin, mitomycin C and carcinogens in cigarettes [10]. This covalent binding can prevent DNA separation, thereby affecting the replication and transcription process in cells and leading to chromosome rearrangement or breakage [11]. In yeast, the researchers found that the Snm1/Pso2 gene is essential for ICL repair [12]. Three SNM1 orthologs, DCLRE1A (SNM1A), DCLRE1B (SNM1B/APOLLO), and DCLRE1C (ARTEMIS), were found in mammalian cells [13, 14]. DCLRE1B, a 60 kDa protein with intrinsic 5' to 3' exonuclease activity, significantly influences telomere maintenance and ICL repair. Nonetheless, whether DCLRE1B contributes to the oncogenesis of particular tumor types is still unknown. As a result, a pan-cancer analysis of DCLRE1B was performed herein.

In our research, we first identified DCLRE1B expression using the TIMER and GEPIA databases. The findings revealed that DCLRE1B was substantially overexpressed in the majority of cancer types including PAAD, which was verified at the protein and mRNA levels using PCR, WB, and IHC analysis. In addition, we also found that DCLRE1B knockdown substantially attenuated cell proliferation and migration in vitro as assessed by CCK-8 and wound healing assay. These findings suggest that DCLRE1B does, in fact, promote oncogenesis and tumor development in human cancers.

The link between DCLRE1B expression and outcome was then investigated. As described previously, in many other cancer types such as ACC, KICH, KIRP, LAML, LGG, LIHC, MESO, PAAD, and SARC, high DCLRE1B expression was linked to a poor prognosis, demonstrating the potential of DCLRE1B as a pan-cancer biomarker for prognosis. TME is a complex ecosystem that contains not only tumor cell populations, but also a variety of mesenchymal cells such as fibroblasts, endothelial cells, normal epithelial cells, mesenchymal stem cells and immune cells [15]. Among them, cancer-associated fibroblasts (CAFs) are important components of TME, located in or around tumor tissues, and are important cells involved in tumor progression and metastasis [16–18]. Studies have found that many malignant tumors, such as prostate cancer, lung cancer, breast cancer, gastric cancer, colorectal cancer and pancreatic cancer, contain a large amount of CAFs [19, 20]. CAFs are particularly high in breast and pancreatic cancer, up to 80% of tumor weight [21]. CAFs are heterogeneous and complex. They can secrete a variety of cytokines, chemokines and growth factors, participate in tumor invasion, growth and metastasis, and promote tumor resistance to treatment. In our research, we also explored the role of tumor-associated fibroblasts in the tumor microenvironment, including pancreatic cancer. The result exhibited that DCLRE1B expression was positively associated with tumor-associated fibroblasts in ACC, KICH, KIRC, KIRP, PAAD, PCPG, THCA, and UCS using Tumor Immune Dysfunction and Exclusion (TIDE) algorithm, that strongly suggests that DCLRE1B might act as a therapeutic target for these cancers. The correlation between DCLRE1B and immune checkpoints has also been explored in this work. It was discovered that DCLRE1B expression had a positive link to ICP genes in many types of cancer, especially in PAAD, in which the correlation coefficients of DCLRE1B with PD-1, PD-L1, PD-L2, and CTLA4 were 0.358, 0.577, 0.593 and 0.378, respectively.

N6-methyladenosine(m^6^A) is the most prevalent epigenetic modification in RNA [22]. In addition to regulating biological processes such as RNA cleavage, transport, translation, and degradation, m6A modification is also crucial in a number of malignancies [23–25]. At present, a number of studies have demonstrated that m6A methyltransferase like 3 (METTL3) is dysregulated in a variety of human malignant tumors and has carcinogenicity [26–28]. Yang et al. [29] demonstrated that knockdown of METTL3 could effectively inhibit the proliferation, migration and invasion of gastric cancer cells, and overexpression of METTL3 increased its carcinogenic function. Some studies have also proved that the up-regulation of METTL3 is one of the reasons for the abnormal modification of m6A in colorectal cancer, and it is positively correlated with tumor metastasis [30]. In addition, METTL3 has also been reported to be involved in pancreatic cancer invasion and metastasis, and is significantly associated with chemotherapy and radiotherapy resistance [31–33]. In our study, we found that there are a large number of m6A modification sites in the CDS region of DCLRE1B by the SRAMP database, indicating that DCLRE1B may be regulated by m6A modification of METTL3. Furthermore, after knockdown of METTL3, we discovered that the expression of DCLRE1B mRNA was likewise dramatically reduced, which further verified our conjecture. More importantly, MeRIP-qPCR findings further established that the m6A modification level of DCLRE1B was substantially diminished after METTL3 knockdown.

To illustrate the potential function of DCLRE1B in cancer, GSEA analysis in PAAD was conducted. And the results showed that DCLRE1B might participate in the regulation of the JAK-STAT signaling pathway. In addition, Fig. 6D exhibited that DCLRE1B was substantially positively linked to PD-L1. Therefore, we considered that DCLRE1B may affect the advancement of pancreatic cancer by mediating the STAT3/PD-L1 signaling pathway. The STAT3/PD-L1 signaling pathway has been reported in multiple studies. A study by Xie et al. demonstrated that apatinib caused non-small cell lung cancer to undergo autophagy and apoptosis by regulating VEGFR2/STAT3/PD-L1 signaling [34]. Another research indicated that Ataxia Telangiectasia Mutated (ATM) could regulate PD-L1 expression in cisplatin-resistant cells by activating JAK/STAT3 signaling [35]. A recent study showed that blockade of lncRNA PVT1 reduced the expression of PD-L1 through the JAK2/STAT3 pathway, thereby inhibiting cell proliferation and invasion in cisplatin resistant epithelial ovarian cancer [36]. The expression of STAT3 and PD-L1 after DCLRE1B knockdown by PCR and western blotting was also explored in this research. According to the findings, STAT3/PD-L1 mRNA and protein expression were substantially down-regulated following DCLRE1B knockdown. According to the above findings, DCLRE1B may play a regulatory role in the proliferation and migration of PC cells via the STAT3/PD-L1 signaling pathway.

## Conclusion

In conclusion, we first explored the differential expression and prognostic value of DCLRE1B in pan-cancer. Furthermore, we confirmed that DCLRE1B may play a role in tumor immune response regulation, which is expected to be a target of tumor immunotherapy. Finally, our study suggests that DCLRE1B may be regulated by METTL3-mediated m6A modification. It can be concluded that DCLRE1B is a key molecule mediating tumorigenesis and development, and may become an important target for tumor therapy, which may open a new path for tumor immunotherapy. DCLRE1B molecular inhibitors or activators still need to be further developed.

### Supplementary Information

**Additional file 1.** **Table S1.** Primers used for qRT‐PCR analysis.

**Additional file 2. Supplementary Figure S1.** The correlation of DCLRE1B expression with tumor stage and grade by the TISIDB website. **Supplementary Figure S2.** The prognostic value of DCLRE1B assessed by Kaplan-Meier Plotter database. (A) The correlation between overall survival (OS) and DCLRE1B expression. (B) The correlation between disease-free survival (DFS) and DCLRE1B expression in pan-cancer. **Supplementary Figure S3.** Associations of DCLRE1B gene expression with sensitivity to chemotherapy (IC50) based on the CellMiner database. IC50, half maximal inhibitory concentration. **Supplementary Figure S4.** Relationship between DCLRE1B expression and m6A-related genes depicted in a heatmap.*P<0.05; **P<0.01; ***P<0.001. **Supplementary Figure S5.** m6A modification sites of DCLRE1B were predicted by SRAMP website. **Supplementary Figure S6.** The PPI network for DCLRE1B using the online GeneMANIA tool.

## Data Availability

The datasets downloaded for supporting the results of this article are publicly available from TCGA (https://portal.gdc.cancer.gov/) and GEO (https://www.ncbi.nlm.nih.gov/geo/).
