# Supplementary material for: DCLRE1B promotes tumor progression and predicts immunotherapy response through METTL3-mediated m6A modification in pancreatic cancer
Source: BMC Cancer. 2023 Nov 7;23:1073. doi: 10.1186/s12885-023-11524-8 (PMC10629169; doi:10.1186/s12885-023-11524-8)
Supplement: Supplementary file 1 — Additional file 1. Table S1. Primers used for qRT‐PCR analysis. [file 12885_2023_11524_MOESM1_ESM.docx]

**Table S1** Primers used for qRT‐PCR analysis

| **Gene** | **Direction** | **Sequences (5′–3′)** |
| --- | --- | --- |
| 18s | Forward | AACCCGTTGAACCCCATT |
| 18s | Reverse | CCATCCAATCGGTAGTAGCG |
| DCLRE1B | Forward | TTGCATCGTCACCTACAGGTAT |
| DCLRE1B | Reverse | ATCGAGGAGGGTTACGGTCA |
| PD-L1 | Forward | GGCATTTGCTGAACGCATTT |
| PD-L1 | Reverse | ACAATTAGTGCAGCCAGGTCT |
| STAT3 | Forward | GAGAGTCAAGATTGGGCATATGC |
| STAT3 | Reverse | GCAATCTCCATTGGCTTCTCAAG |
| METTL3 | Forward | TTGTCTCCAACCTTCCGTAGT |
| METTL3 | Reverse | CCAGATCAGAGAGGTGGTGTAG |
