# Supplementary material for: DCLRE1B promotes tumor progression and predicts immunotherapy response through METTL3-mediated m6A modification in pancreatic cancer
Source: BMC Cancer. 2023 Nov 7;23:1073. doi: 10.1186/s12885-023-11524-8 (PMC10629169; doi:10.1186/s12885-023-11524-8)
Supplement: Supplementary file 2 — Additional file 2. Supplementary Figure S1. The correlation of DCLRE1B expression with tumor stage and grade by the TISIDB website. Supplementary Figure S2. The prognostic value of DCLRE1B assessed by Kaplan-Meier Plotter database. (A) The correlation between overall survival (OS) and DCLRE1B expression. (B) The correlation between disease-free survival (DFS) and DCLRE1B expression in pan-cancer. Supplementary Figure S3. Associations of DCLRE1B gene expression with sensitivity to chemotherapy (IC50) based on the CellMiner database. IC50, half maximal inhibitory concentration. Supplementary Figure S4. Relationship between DCLRE1B expression and m6A-related genes depicted in a heatmap.*P<0.05; **P<0.01; ***P<0.001. Supplementary Figure S5. m6A modification sites of DCLRE1B were predicted by SRAMP website. Supplementary Figure S6. The PPI network for DCLRE1B using the online GeneMANIA tool. [file 12885_2023_11524_MOESM2_ESM.docx]

**
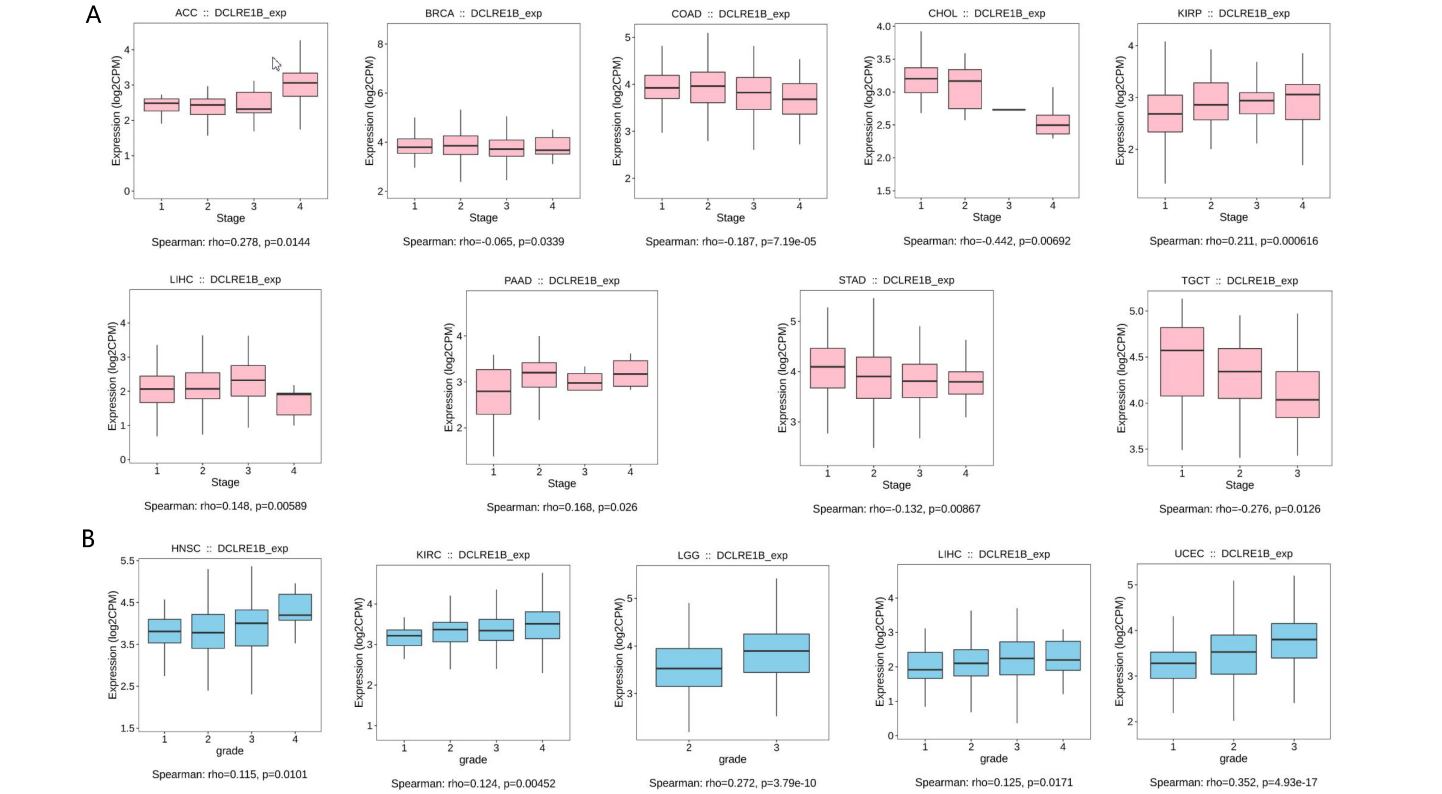
**

**Supplementary Figure S1** The correlation of DCLRE1B expression with tumor stage and grade by the TISIDB website.

**
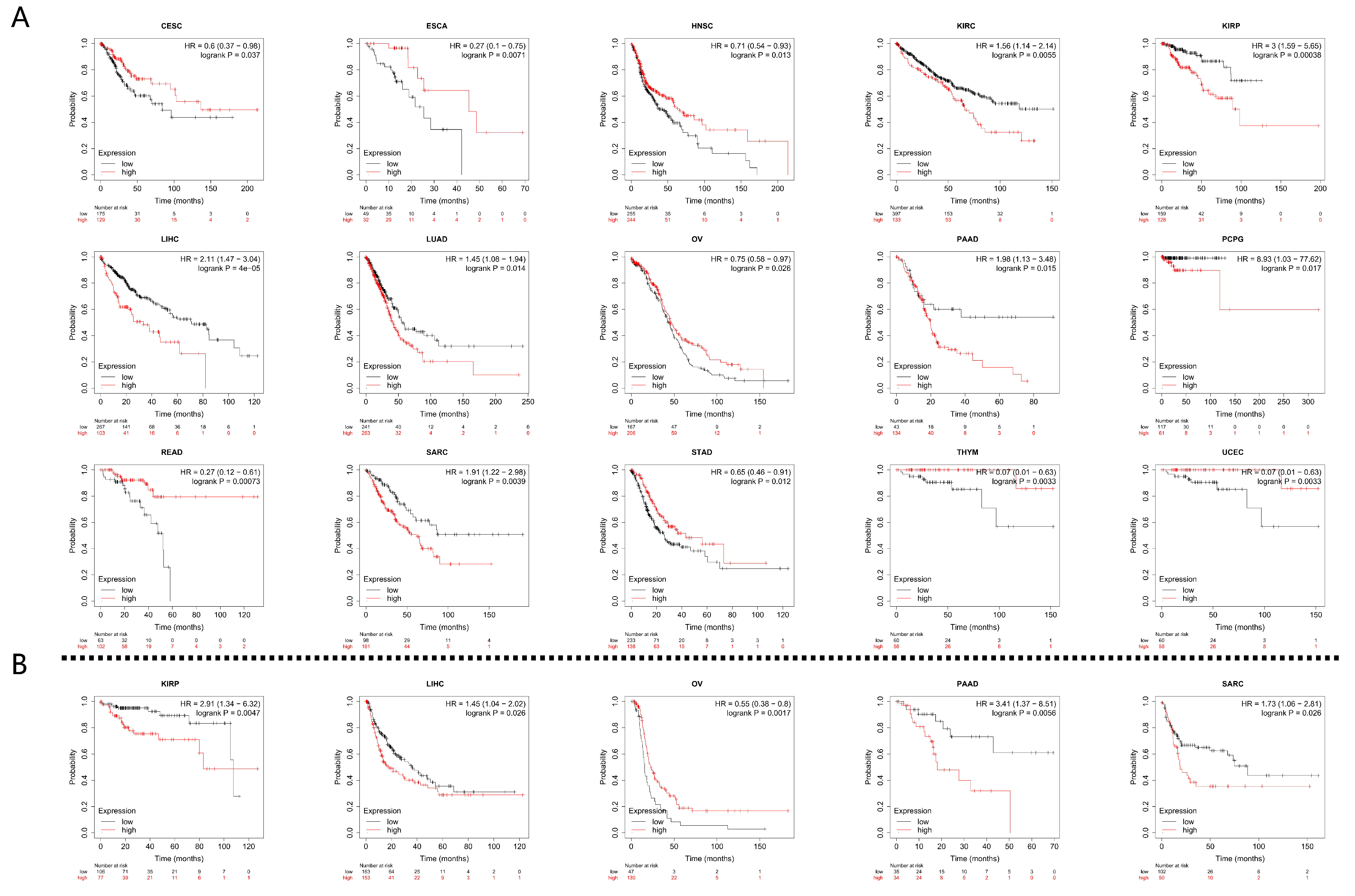
**

**Supplementary Figure S2** The prognostic value of DCLRE1B assessed by Kaplan-Meier Plotter database. (A) The correlation between overall survival (OS) and DCLRE1B expression. (B) The correlation between disease-free survival (DFS) and DCLRE1B expression in pan-cancer.

**

**

**Supplementary Figure S3** Associations of DCLRE1B gene expression with sensitivity to chemotherapy (IC50) based on the CellMiner database. IC50, half maximal inhibitory concentration.

**
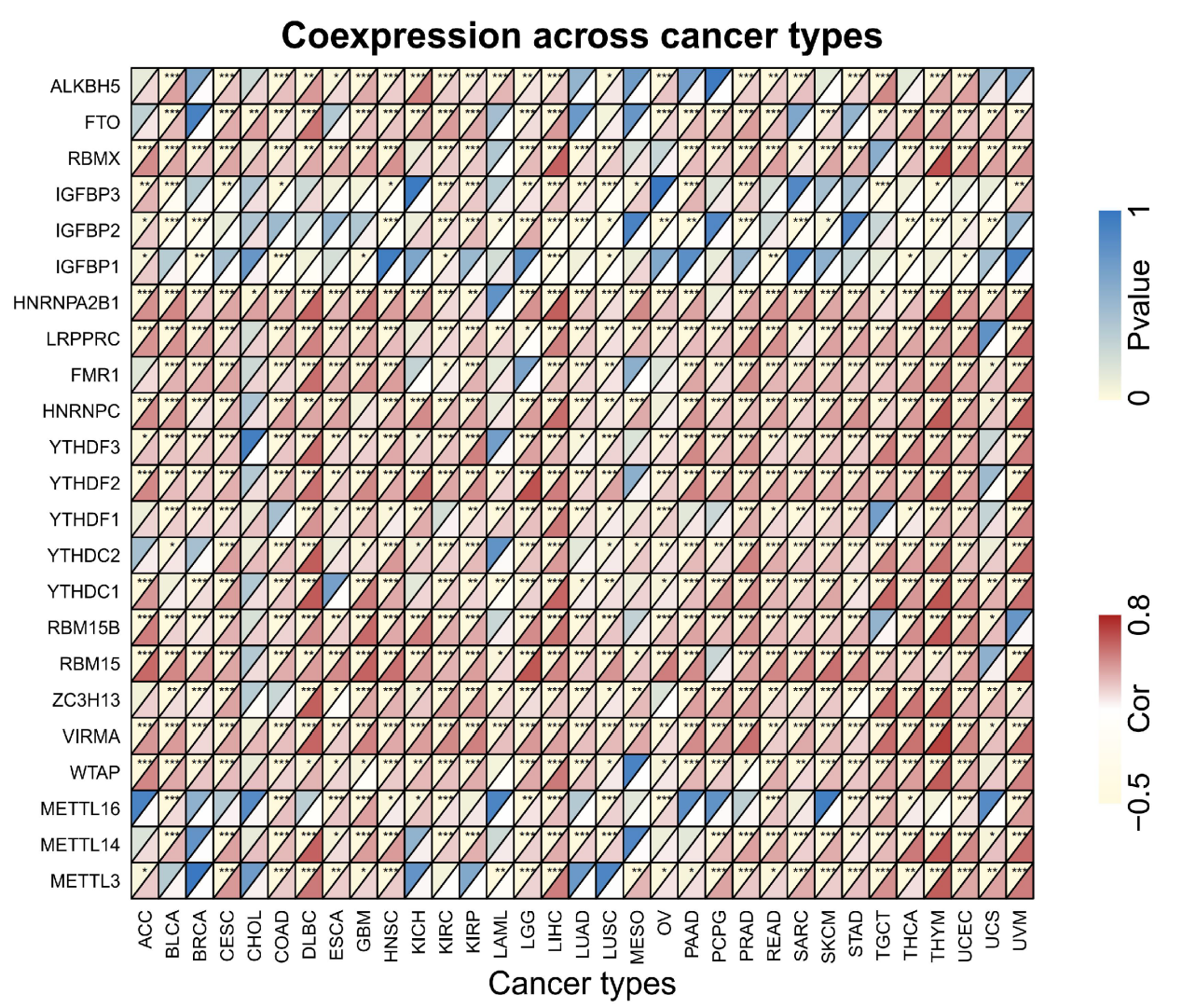
**

**Supplementary Figure S4** Relationship between DCLRE1B expression and m6A-related genes depicted in a heatmap. *P<0.05; **P<0.01; ***P<0.001.

**
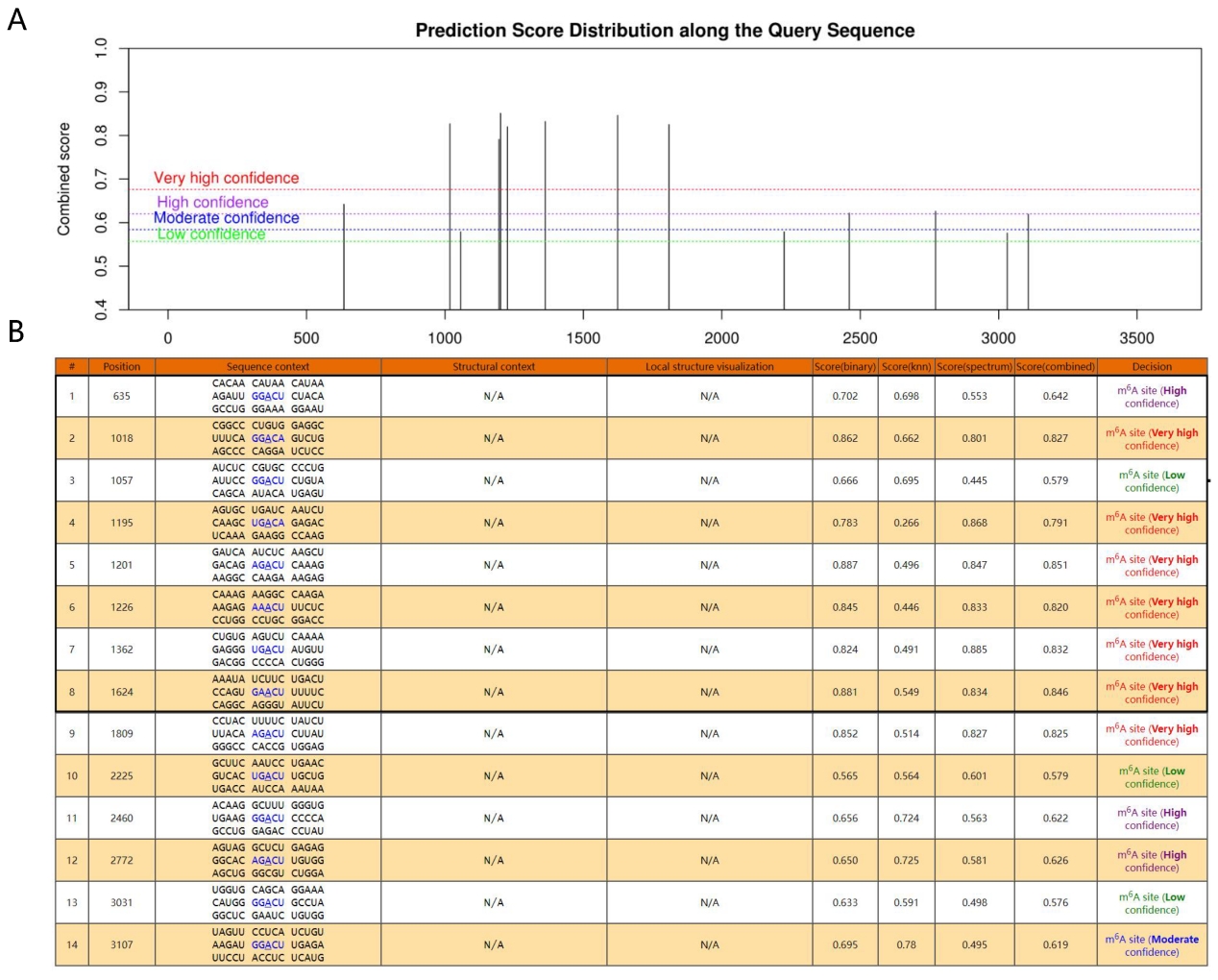
**

**Supplementary Figure S5** m6A modification sites of DCLRE1B were predicted by SRAMP website.

**
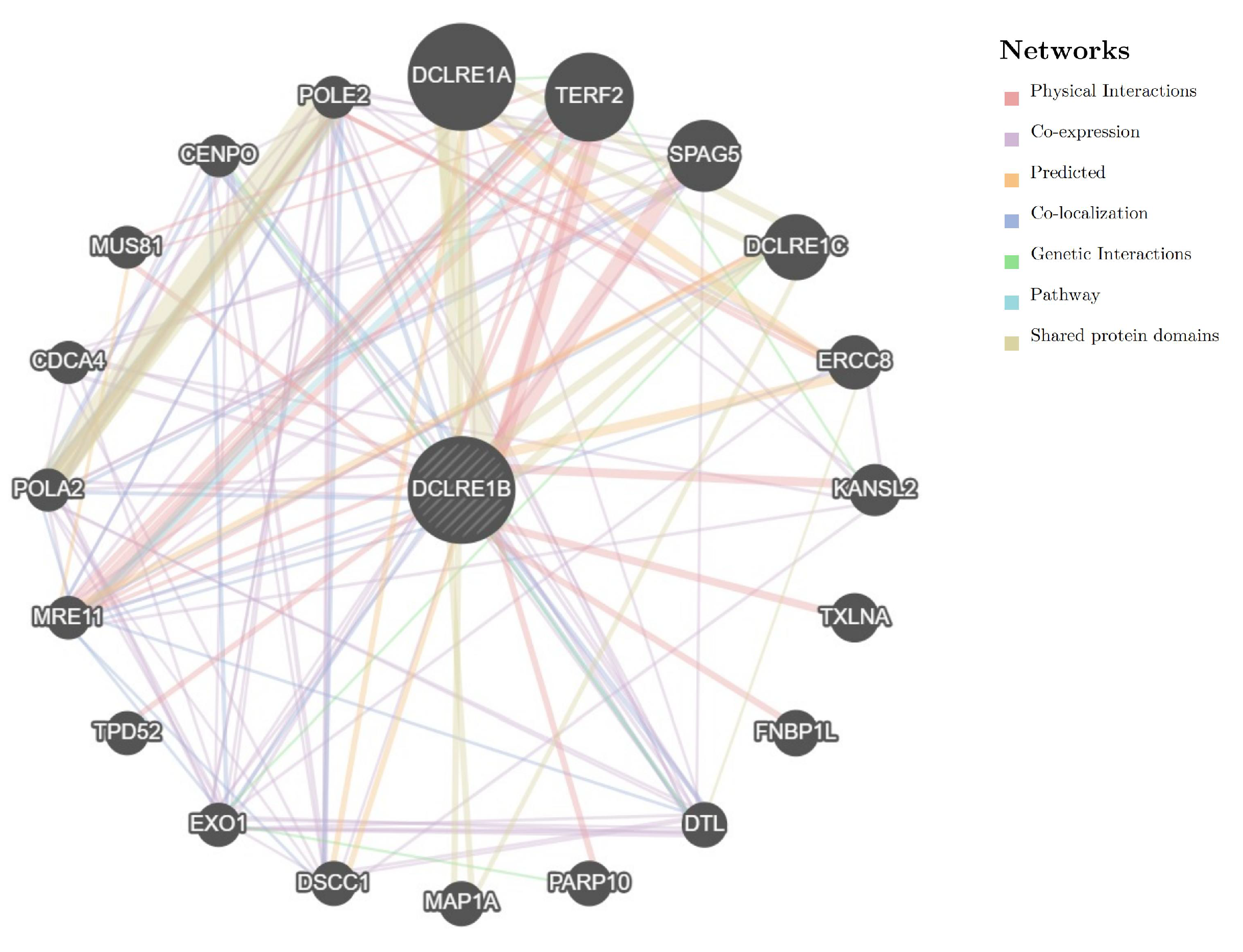
**

**Supplementary Figure S6** The PPI network for DCLRE1B using the online GeneMANIA tool.
